# Prosaposin variants in sporadic, familial, and early-onset Parkinson's disease: a Taiwanese case–control study and meta-analysis

**DOI:** 10.1038/s41598-024-51646-y

**Published:** 2024-01-26

**Authors:** Ming-Che Kuo, Yung-Tsai Chu, Yu-An Su, Meng-Ling Chen, Ruey-Meei Wu

**Affiliations:** 1 Department of Neurology, Centre for Parkinson and Movement Disorder, National Taiwan University Hospital, Taipei, Taiwan; 2 Department of Medicine, National Taiwan University Cancer Center, Taipei, Taiwan; 3 Graduate Institute of Clinical Medicine, College of Medicine, National Taiwan University, Taipei, Taiwan; 4 Department of Neurology, National Taiwan University Hospital Jinshan Branch, New Taipei City, Taiwan; 5 Genome and Systems Biology Degree Program, Academia Sinica and National Taiwan University, Taipei, Taiwan; 6 Department of Neurology, College of Medicine, National Taiwan University, No.7, Chung Shan South. Road, Taipei, 10002 Taiwan

**Keywords:** Clinical genetics, Genetic association study, Parkinson's disease, Neurology

## Abstract

Polymorphisms in the *PSAP* gene, which encodes prosaposin and is involved in the lysosomal function, yielded conflicting results regarding the association with Parkinson’s disease (PD). Therefore, this study aims to investigate the role of *PSAP* in familial PD (FPD), early onset PD (EOPD) with age at onset before 50 years old, and sporadic PD (SPD) among Taiwanese population, and summarize relevant studies via meta-analysis. By sequencing exon 1 to 14 in 183 FPD and 219 EOPD, two novel exonic variants were found in EOPD, including p.A146E (c.437C > A) on exon 5 and p.Y248C (c.743A > G) on exon 7. Furthermore, four previously reported intronic variants (rs142614739/rs74733861), rs749823, rs4747203 and rs885828) in intron 11 and 12 were analyzed in 485 SPD and 712 in-hospital controls, in addition to the aforementioned FPD and EOPD groups. The adjusted odd ratios (ORs) by age and sex, only rs142614739 was significantly associated with higher risk of EOPD (OR = 1.85, 95% CI = 1.33–2.58). The risk effect was further confirmed by the meta-analysis of the association between rs142614739 and the risk of PD in both common effect (OR = 1.29, 95% CI = 1.11–1.50) and random effect (OR = 1.29, 95% CI = 1.11–1.50). Our findings suggest that the *PSAP* rs142614739 variant is associated with the risk of EOPD. Further functional studies are warranted to elucidate the biochemical mechanisms.

## Introduction

Parkinson's disease (PD) is a neurodegenerative disease presenting with bradykinesia, rigidity, tremor, and gait disturbance as motor features and is one of the most common movement disorders in people older than 65 years^1^. Although most of the cases are sporadic, it is estimated that 3–5% of patients have monogenic causes linked to known Parkinson's disease genes, while an additional 16–36% of patients have genetic risk variants that increase the risk of PD^1^. The pathophysiological mechanisms of those genes mostly involve excessive pathological aggregation of alpha-synuclein, mitochondrial dysfunctions, or deficits in lysosomal degradation to maintain cellular homeostasis^2,3^. A well-known mutation associated with significant PD risk is the mutations in the glucocerebrosidase gene (*GBA1*), leading to reduced lysosomal enzyme activity and build-up of glucocerebrosides^4^. Recently, a lysosome-related gene, *PSAP,* has also been reported to have an association with PD risk^5,6^.

The *PSAP* gene encodes the prosaposin protein and includes 14 exons. After translation, prosaposin is cleaved into 4 active units, saposin A to D, which serve as sphingolipid activators required for certain lysosomal enzymes^7^. For example, saposin C can activate glucocerebrosidase, which is encoded by *GBA1*. Mutations in saposin C and B domain of *PSAP* are implicated in Gaucher’s disease and metachromatic leukodystrophy, respectively^8,9^. Notably, several pathogenic mutations in the saposin D domains of *PSAP* were firstly reported in three families with autosomal dominant inheritance in PD^6^. They further analyzed the saposin D domain, which is encoded in exon 10 to 14, in a combined cohort enrolling 440 Japanese and 705 Taiwanese patients with sporadic PD (SPD). Notably, two intronic variants, rs885828 in intron 12, and rs4747203 in intron 11, were found to be associated with an increased risk of SPD.

Later, more studies have been conducted to investigate the association between *PSAP* variants and risk of PD. However, the results were conflicting. No significant associations of rs885828 and rs4747203, intronic variants, have been reported using genome-wide association studies and Sanger sequencing of *PSAP* exon 10–14 in SPD^10,11^. On the other hand, Lin et al*.* discovered a protective effect of these two intronic variants^12^. Chen et al*.* also reported a significantly reduced risk of PD in patients of SPD who carried *PSAP r*s4747203 variant^13^.

Based on aforementioned inconsistent results regarding the effect of *PSAP* intronic variants on the risk of PD, this study aims to clarify the role of *PSAP* variants in different subgroups of PD among Taiwanese population by enrolling patients with early-onset PD (EOPD), who have the onset age of PD before 50 years old, and patients with familial PD (FPD), who have a family history of PD, patients with sporadic PD (SPD), who have no family history and have disease onset older than 50 years of age, and health controls (HC). Both exonic variant analysis and intronic variant association studies were utilized. The first part of the study looks at exonic mutations of *PSAP* in EOPD and FPD, in comparison with HC from a public database. The second part of the study looks at intronic variants in EOPD, FPD and SPD, in comparison with HC from NTUH. Meta-analysis is also conducted to summarize the current findings on the association between *PSAP* variants and risk of PD.

## Results

### Demographic characteristics

A total of 887 patients, including 485 SPD, 219 EOPD, and 183 FPD (117 ADPD, 66 ARPD) were enrolled. A total of 711 HC, including 300 from Taiwan Biobank and 412 from NTUH were also enrolled. The mean age, gender proportion, disease onset, and disease status were summarized in Table 1. The mean age at examination was 58.9 ± 11.1 years in HC, 67.0 ± 4.5 years in SPD, 46.7 ± 7.5 years in EOPD and 63.2 ± 11.0 in FPD. The mean age at disease onset was 62.4 ± 3.4 years in SPD, 41.4 ± 8.1 years in EOPD and 58.7 ± 12.3 in FPD. The median Hoehn and Yahr (H&Y) scale was 2 in all PD subgroups.

**Table 1:** Participant demographics.

	Control	EOPD	SPD	FPD
Number	712	219	485	183
Age at exam (years)	58.9 ± 11.1	46.7 ± 7.5	67.0 ± 4.5	63.2 ± 11.0
Gender (male, %)	281 (39.4)	110 (50.2)	242 (49.9)	107 (58.5)
Age at onset (years)	NA	41.4 ± 8.1	62.4 ± 3.4	58.7 ± 12.3
H&Y stage (median, interquartile range)	NA	2 (1–2)	2 (2–3)	2 (2–2)

*EOPD* Early-onset Parkinson’s disease; *SPD* Old-onset sporadic Parkinson’s disease; *FPD* Familial Parkinson’s disease; *H&Y* Hoehn and Yahr.

### Mutation analysis of *PSAP*

Exonic mutations of *PSAP* in EOPD and FPD patients were compared with HC from community-based public datasets including gnomAD EAS (East Asia population) and Taiwan Biobank. Three pathogenic exonic variants (p.C412Y, p.Q453P, p.C451_L477del) were not found in EOPD and FPD groups^6^. Two novel exonic variants were identified, including p.A146E on exon 5 (c.437C > A), and p.Y248C on exon 7 (c.743A > G), in 2 cases of EOPD. Those two variants are not reported in public datasets. Based on the multiple sequence alignment analysis with other species, most of them were shown to be evolutionarily conserved amino acid positions in PSAP (Supplementary Fig. S1). According to the criteria of the American College of Medical Genetics, the variant of p. Y248C variant is classified as likely pathogenic variants and the variant of p.A146E is classified as variants of uncertain significance^14^. We were unable to perform the co-segregation analysis of these two exonic variants in proband’s family due to noncompliance and loss follow up. All variants identified in our cohort are provided in Supplementary Table S1.

### Genotypic and allelic analysis of 4 intronic variants around Saposin D domain

The Chi-Square test for Hardy–Weinberg equilibrium were all non-significant in all four intronic variants (*p*-value 0.42 in rs4747203, 0.11 in rs885828, 0.67 in rs142614739 and 0.85 in rs749823). The allelic and genotypic frequency of 4 intronic variants (adjusted by age and sex), rs4747203, rs142614739/rs74733861 (this variant is referred to rs142614739 in the following text), rs749823 and rs885828, between each group of PD patients and HC were compared respectively (Table 3). The results of genotypic and allelic frequencies of rs4747203, rs142614739, rs749823 and rs885828 in each group were shown in Table 2. In the logistic regression adjusting age and sex, the rs142614739 was significantly associated with increased disease risk in EOPD by additive model (odds ratio [OR] = 1.85, 95% confidence interval [95% CI]) = 1.33–2.58, *p* < 0.001). There was no statistically significant association of rs4747203 and rs885828 between total and other subgroups of PD patients and HC, although there was a protective trend of rs885828 variants in EOPD (OR = 0.77, 95% CI = 0.60–0.99, *p* = 0.04). Also, the additive models of rs4747203 showed a statistically non-significant protective effect in EOPD (OR = 0.79, 95% CI = 0.62–1.02, *p* = 0.07).

**Table 2:** The genotypic, dominant, and recessive genotypes and allelic frequency of four intronic variants between patients with different subgroups of Parkinson’s disease and healthy controls.

Variants	Genotype/Allele	Association test	Control*	EOPD*	SPD*	FPD*
rs4747203	Genotype	Genotypic (TT/TC/CC)	97/343/272	40/104/74	75/203/201	21/93/69
	Allele (major/minor)	T/C	536/886	184/252	353/605	135/231
rs142614739	Genotype	Genotypic (wt wt/wt del^#^/del del)	509/184/14	115/84/5	333/130/8	132/47/4
	Allele (major/minor)	wt/del	1202/212	314/94	796/146	311/55
rs749823	Genotype	Genotypic (CC/CT/TT)	382/228/51	93/54/14	274/167/23	56/53/5
	Allele (major/minor)	C/T	992/330	240/82	715/213	165/63
rs885828	Genotype	Genotypic (CC/CT/TT)	85/314/272	39/104/75	77/205/203	20/77/69
	Allele (major/minor)	C/T	484/858	182/254	359/611	117/215

*Some data for controls, EOPD, FPD, and SPDcases are missing due to lack of genotypic data.

^#^AGTCTC deletion.

*EOPD* Early-onset Parkinson’s disease; *FPD* Familial Parkinson’s disease; *PD* Parkinson’s disease; *SPD* Old-onset sporadic Parkinson’s disease; *wt* Wild-type (without AGTCTC deletion).

### Meta-analysis of 4 intronic variants

**Figure 1:**
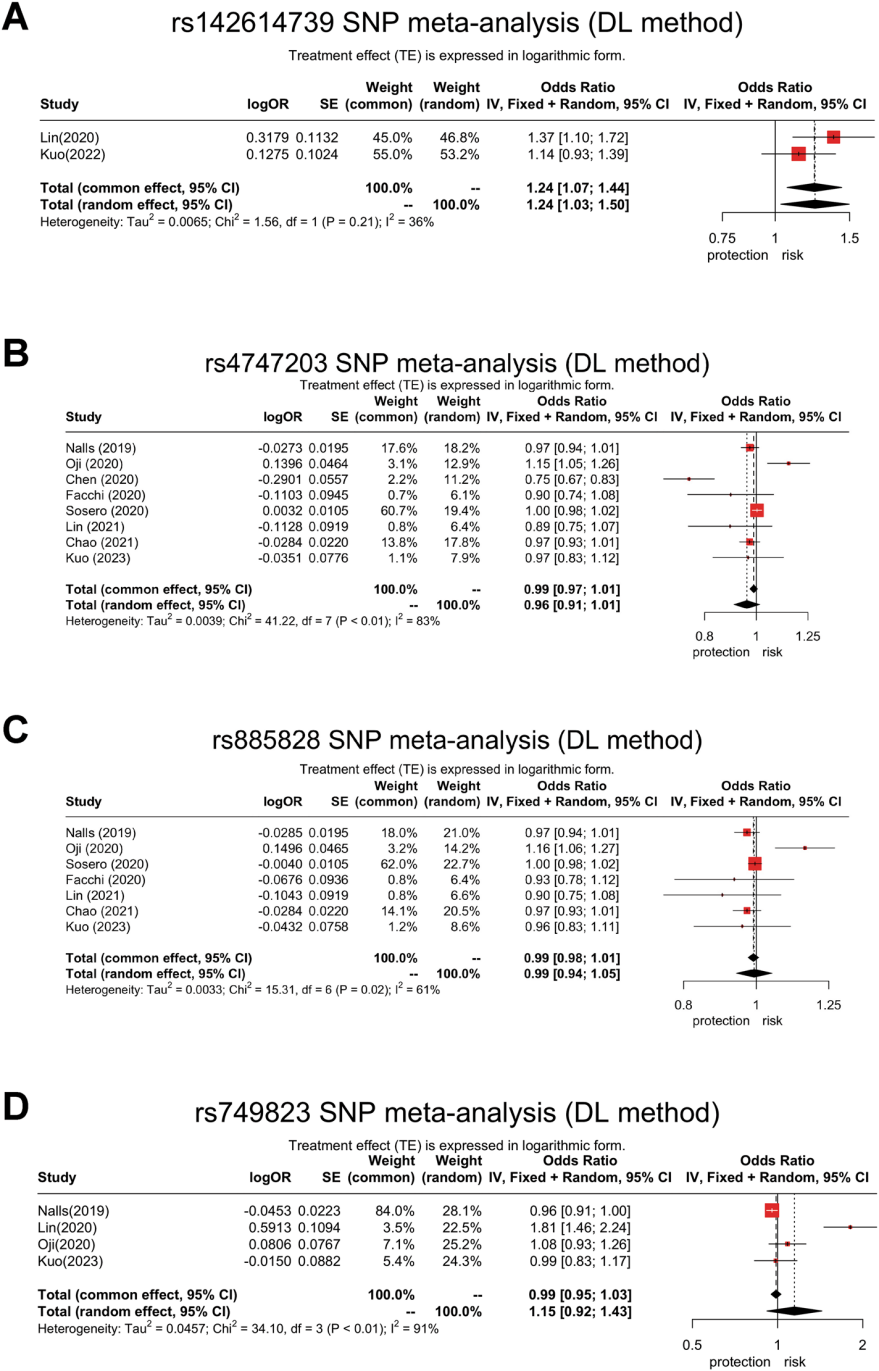
Meta-analysis of the effects of rs142614739, rs4747203, rs885828 and rs749823 on the risk of Parkinson’s disease: rs142614739 was significantly associated with the risk of PD in both common effect (OR = 1.24, 95% CI = 1.07–1.44) and random effect (OR = 1.24, 95% CI = 1.03–1.50) in patients with total PD including EOPD, SPD, and FPD (**A**). In contrast, rs4747203 (common effect: OR = 0.99, 95% CI = 0.97–1.01; random effect: OR = 0.96, 95% CI = 0.91–1.01), rs885828 (common effect: OR = 1.00, 95% CI = 0.98–1.01; random effect: OR = 1.00, 95% CI = 0.94–1.05) and rs749823 (common effect: OR = 0.99, 95% CI = 0.95–1.03; random effect: OR = 1.23, 95% CI = 0.92–1.43) were not associated with the risk of PD (**B**–**D**).

Pooled analysis of the effects of rs142614739, rs885828, rs4747203 and rs749823 on the risk of PD was performed after incorporating multiple studies (Fig. 1). The sample size of each study was summarized in Supplementary Table S3. As shown in Fig. 1A, rs142614739 was significantly associated with the risk of PD (common effect OR = 1.24, 95% CI = 1.07–1.44; random effect OR = 1.24, 95% CI 1.03–1.50). In contrast, rs885828, rs4747203 and rs749823 were not significantly associated with the risk of PD (Fig. 1B–D).

## Discussions

In this study, we performed a comprehensive sequencing of 14 exons in *PSAP* and discovered 2 novel PD-related exonic variants in patients with FPD. Furthermore, we investigated 4 intronic variants around saposin D domain and we found that rs142614739 significantly increased the risk of younger-onset (< 50 years old) PD patients (Table. 3, Fig. 1) rather than in patients with sporadic PD (onset age > 50 years-old) or familial PD (positive family history). The meta-analysis further confirmed the risk effect of rs142614739 and concluded the neutral effect of the previously reported intronic variants (rs885828, rs4747203, and rs749823).

**Table 3:** The association tests of each variant in the risk of Parkinson’s disease by genotypic and allelic frequency analysis.

Variants	Genotype/Allele	Total PD		EOPD		SPD		FPD	
		OR (95% CI)	*p*-value	OR (95% CI)	*p*-value	OR (95% CI)	*p*-value	OR (95% CI)	*p*-value
rs4747203	Additive model (CC/TC/TT)	0.99 (0.86–1.15)	0.97	0.79 (0.62–1.02)	0.07	1.06 (0.89–1.27)	0.49	1.08 (0.84–1.39)	0.54
rs142614739	Additive model (del# del/wt del/wt wt)	1.14 (0.93–1.39)	0.21	**1.85 (1.33–2.58)**	< 0.001*	0.79 (0.31–2.01)	0.62	0.97 (0.70–1.36)	0.87
rs749823	Additive model (TT/TC/CC)	0.99 (0.83–1.17)	0.87	1.10 (0.81–1.49)	0.57	0.91 (0.74–1.12)	0.37	1.12 (0.81–1.54)	0.48
rs885828	Additive model (TT/TC/CC)	0.96 (0.83,1.11)	0.57	0.77 (0.60–0.99)	0.04	1.01 (0.85–1.21)	0.89	1.07 (0.82–1.39)	0.52

Age- and sex- adjusted odds ratios and 95% confidence interval from the logistic regression analyses by Wald test compared with controls.

*Significant after Bonferroni correction (0.05/4 = 0.0125).

^#^AGTCTC deletion.

*CI* Confidence interval; *EOPD* Early-onset Parkinson’s disease; *FPD* Familial Parkinson’s disease; *OR* Odds ratio; *SPD* Old-onset sporadic Parkinson’s disease; wt, Wild-type (without AGTCTC deletion).

Significant values are in bold.

The biogenesis of saposin A-D cleaved from full-length pro-peptide Prosaposin presented in endosomes intracellularly^7^. In particular, saposin C can activate glucocerebroside in lysosome, which is linked to the pathogenesis of PD^3^. Moreover, *the PSAP* gene was found to be upregulated in the substantia nigra of PD versus controls^15^. Previous evidence implies that any deficit in the functional chain of prosaposin could potentially impair the endo-lysosomal pathways, and eventually result in PD-associated biochemical aberrances and phenotypes. There were 2 exonic variants disclosed in this study. The missense mutation p. Y248C is located at the saposin B domain. Previous study has shown that functional deficit of saposin B leads to diminished degradation of glycosphingolipids in the brain^16^. The other missense mutation p.A14E is located between saposin A and B domain. We speculated that this mutation may interfere with the peptide splicing of prosaposin or influence other post-translation modification processes. Unfortunately, co-segregation analysis cannot be performed due to loss of follow-up so that the pathogenicity of those variants cannot be further validated. Furthermore, only exons in patients with EOPD and FPD were sequenced while patients with SPD and HC were not. Rare variant burden analysis could not be performed to compare the frequency of rare variant between cases and controls. We could only use the public information from Taiwan Biobank and GnomAD as an alternative to reflect the possible genetic background of general populations. Functional study, such as changed lysosomal burden in the cell model and motor impairment in the animal model, can also be considered in the future^6^.

The role of two intronic variants (rs885828, and rs4747203) remain controversial^6^. However, numerous studies reported neutral or even protective effect in these 2 intronic variants^10–13,17^. A protective effect of rs4747203 was reported and a risk effect of rs142714739^12,13^ was observed. While the largest cohort in the meta-analysis from 23andMe including 1,400,000 HC and 37,688 PD did not show no association of rs4848203, rs885828, and rs749823 variants with the risk of PD (Supplementary Table S3)^18^. It is possible that intronic variants may influence mRNA splicing or post-transcriptional regulation^19^. However, the functional impact of intronic variants in mRNA splicing is still a challenge in the genomic research field^19^. More importantly, although some software is developed to predict the cleavage site of peptides by various proteases in databases^20^, it is still hard to answer whether these intronic variants located between exon 10–11 are able to impair the topology of pre-peptide and/or derived peptides intracellularly. In order to support the genomic impact at the cellular level, the expression level of RNA or protein products may reflect the difference^21^. Facchi et al*.* has found that *PSAP* RNA expression level in the whole blood was significantly correlated with rs885828 genotypes^11^. Although the exact mechanisms that these intronic variants are involved remain to be discovered, these findings provide an alternative method to examine the biological effect of those intronic variants.

In fact, the presence of variants in rs885828 and rs4747203 are in linkage disequilibrium as mentioned by Sosero et al^17^. This phenomenon is more prominent in Caucasian (r2 = 1, D′ = 1) and less commonly seen in Japanese healthy controls (D′ = 0.7) in rs885828^17^. Therefore, ethnic variation in the allele frequency of these two intronic variants between Asian and Caucasian may account for the different analytic results. Interestingly, few except one study mentioned the existence of *PSAP* rs142614739, which is located between rs885828 and rs4747203 at the intron 10–11^12^ and has been merged into rs74733861 in dbSNP and ClinVar Miner^22,23^. This variant is a 6-nucleotide deletion between exons 10 and 11, which may impair the following mRNA splicing or peptide cleavage. Lin et al*.* also found that two out of four common haplotypes, including H2 (rs885828 T allele, rs142614738 wt, rs4747203 G allele) and H4 (rs885828 C allele, rs142614739 del, rs4747203 A allele), were significantly associated with PD^12^. As for the European population, there is a moderate linkage disequilibrium between rs142614739 and rs885828 (R^2^ = 0.3579, D’ = 1) and between rs142614739 and rs4747203 (R^2^ = 0.3579, D′  = 1) in the database of LDpop Tool (https://ldlink.nih.gov/?tab=ldpop). Thus, the significance of rs142614739 in the European population is still uncertain. What we found is that rs142614739 del is associated with EOPD in our cohort and meta-analysis. One interpretation is that this intronic variant could affect the splicing or the stability of mRNA. The other explanation is that those intronic variants are makers of nearby genetic mutation due to linkage disequilibrium.

In the study published by Sosero et al., the variants rs4747203 and rs885828 are not associated with the risk of PD and the age at onset^17^. Nevertheless, we found that rs142614739 was associated with increased risk of EOPD, while the effect is less prominent in sporadic PD older than 50 years old at the onset of symptoms. In the study published by Oji et al., they also mentioned that the included Japanese cohort has significantly younger average age at onset (50.9 ± 13.7 years)^6^. This age difference might explain the different effect of the intronic variant compared with other studies. A larger association study in multi-ethnic populations are needed to determine whether *PSAP* variants are associated with the younger onset of PD.

There are limitations in this study. First, although we enroll patients with different age of onset and presence of family history or not, the overall sample size is relatively small in PD patients which may undermine the statistical power to evaluate the effect of each variant. Second, the female is predominant in our control group, which may indicate more healthy volunteers in our hospital. Previous study has shown that gender may influence the health care-seeking behavior, especially in women participants^24^. However, no obvious gender effect has been reported in the polymorphism distribution of *PSAP* gene before. We also adjust sex and age in statistical analysis. Therefore, we presumed that the gender imbalance may not have a major impact on the results. Third, lack of burden analysis and co-segregation study makes the clinical effect of those rare exonic variants uncertain. Fourthly, although this study aimed to investigate the associations between pathological traits and genetic variations, environmental factors may also influence the risks of developing PD. Thus the results should be carefully interpreted because respective contributions of genetic and environmental factors were not conducted in this study. Lastly, there were also several limitations in the meta-analysis. The age of disease onset was not provided in all studies included in the meta-analysis. Therefore, the association between age of disease onset or other phenotypic traits with *PSAP* variants cannot be analyzed in our meta-analysis. Moreover, rs142614739 has been only published in 2 studies so the overall population size is still low in the meta-analysis. The exact mechanism of these intronic variants remains uncertain and warrants functional studies of *PSAP* in the lysosomal pathway and even alpha-synuclein propagation pathways.

In conclusion, two exonic variants in the *PSAP* gene were highlighted, one of which is possibly pathogenic. The analysis of intronic variants reveals that rs142614739 was associated with significantly increased risk of EOPD, suggesting a potential modification of disease age at onset. On the other hand, rs885828 and rs4747203 do not have a significant effect on the risk of PD in this study and meta-analysis. Further functional study on the mechanism of rs142614739 leading to increased risk of PD is highly warranted.

## Method

### Participants and study design

**Figure 2:**
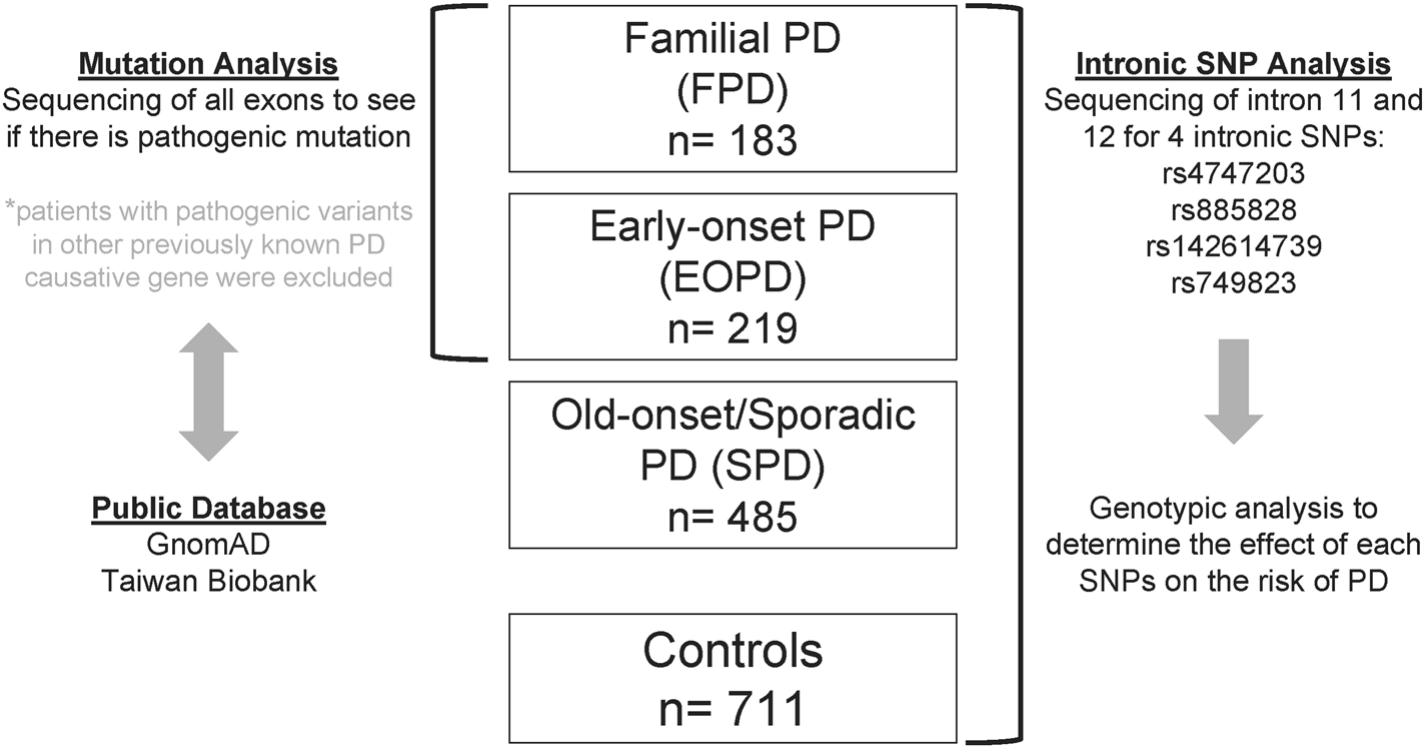
Participants and study design: Three subgroups of Parkinson’s disease (PD) patients, including familial PD (FPD), early onset PD (EOPD) and old-onset/sporadic PD (SPD) were enrolled. Exons 1 to 14 were Sanger sequenced in FPD and EOPD. Exonic and intronic variants we identified were compared with public data on GnomAD and Taiwan Biobank first. Among four intronic variants on intron 11 and 12 on the saposin D domain of PSAP gene, including rs4747203, rs885828, rs142614739, and rs749823, were sequenced in additional cohorts including SPD and both community- and hospital-based controls to compare the genotypic and allelic frequencies between groups.

The study design of this case-control association study is summarized in Fig. 2. Patients with SPD, EOPD, FPD, and HC were enrolled at the Excellent Centre for Parkinson and Movement Disorder, Department of Neurology, National Taiwan University Hospital (NTUH). The diagnosis of PD was based on the UK Parkinson’s Disease Society Brain Bank clinical diagnostic criteria by two movement disorder specialists (M-C Kuo, R-M Wu)^25^. The EOPD is defined as the age at symptom onset before 50 years old. The SPD is classified as the patients who had presentation of parkinsonian motor features at age older than fifty. The FPD is defined as the presence of a positive family history of PD in the proband’s family in either autosomal dominant (AD) or autosomal recessive (AR) patterns. Volunteers or spouses of the diseases were enrolled and examined by doctors or nurses at the Excellent Centre for Parkinson and Movement Disorder, Department of Neurology, NTUH to exclude major systemic or neurodegenerative diseases. All participants provided written informed consent. Ethics approval was obtained from the Ethics Committee of the National Taiwan University Hospital, Taipei, Taiwan.

### DNA preparation and sequencing

Sanger sequencing was performed for *PSAP* exon 1–14 in patients of FPD and EOPD to determine if there is any pathogenic mutation hotspot and *PSAP* intron 11–12 in all PD patients and HC for the analysis of 4 previously-reported intronic variants. In the groups of FPD and EOPD, pathogenic/likely pathogenic variants in other previously known PD causative genes were excluded by multiplex ligation-dependent probe amplification and targeted next-generation sequencing panel^26^. For DNA preparation, 10 ml of venous blood was collected and genomic DNA was extracted by Blood/Cell DNA Maxi Kit (Geneaid GDM025). DNA was then amplified by polymerase chain reaction (PCR). Different primers were used to amplify genomic segments of each *PSAP* exon or intron. Primer sequences are listed in Supplementary Table S2. PCR reactions were performed in a total volume of 25 μl, containing 12.5 μl of PCR Master Mix (Cat.No: M7502, Promega), 9.5 μl nuclease-free water, 10 μM of forward-primer, 10 μM of reverse primer, and 200 ng of genomic DNA. PCR conditions were 2 min 30 s of initial denaturation at 95 °C, and 35 cycles of 30 s of denaturation at 95 °C, 30 s at annealing temperature, and 35 s of extension at 72 °C. PCR products were purified using Presto™ 96 Well PCR Cleanup Kit (96DFH10, Geneaid). Direct sequencing was performed using BigDye™ Terminator chemistry (version 3.1; Life Technologies) as recommended by the manufacturer. Then DNA was precipitated by ethanol and loaded on an Applied Biosystem 3730 DNA Analyzer (Life Technologies). For sequencing analysis, Chromas and Unipro UGENE were used.

### Exonic mutation analysis

Mutations were compared with the public reference database, including gnomAD (https://gnomad.broadinstitute.org/) and Taiwan Biobank (https://taiwanview.twbiobank.org.tw/variant.php)^27^. Variants identified were annotated according to GenBank accession number NM_002778, corresponding to Homo sapiens *PSAP*, transcript variant 1. For the analysis of exons in *PSAP*, missense variants were only considered deleterious when they were predicted to be damaging by SIFT and PolyPhen-2. Rare variants were further classified as pathogenic, likely pathogenic, variant of uncertain significance (VUS), or likely benign or benign, according to the recommendations of the American College of Medical Genetics^14^. Amino acid sequence comparison across different species is done to examine the evolutionary conservation via multiple sequence alignment by constraint-based alignment tool for multiple protein sequences (COBALT)^28^.

### Statistical analysis

Demographics of participants in each group are summarized in mean ± standard deviation for continuous variables and median ± interquartile range for the H&Y scale. Chi-square test was used to test for the Hardy–Weinberg equilibrium of four intronic variants in the controls. To adjust age and sex, multivariate logistic regression incorporating age, sex and each variant status was used. Additive inheritance model (genotype AA as 2, AB as 1 and BB as 0) was used in the regression. The Wald test was applied to test for the significance of the genotype variable. We used Bonferroni correction to adjust for multiple testing for four variants, after which a *p*-value less than 0.0125 (0.05 divided by 4) was regarded as statistically significant, as detailed in a previous study^27^. The strength of the association between the variants and Parkinson’s disease susceptibility was evaluated by OR.

### Meta-analysis

The publications were searched from the databases of Medline, PubMed, and ISI Web of Knowledge. The last search was updated on Oct, 31, 2023. The search terms “prosaposin gene”, “saposin”, “Parkinson’s disease”, “SNPs” and “polymorphism” were used. The references of associated publications were carefully evaluated to obtain as many as possible. Only those published studies with full-text articles in English were included in this meta-analysis. For overlapping and republished studies, only the first one with the largest samples was included. The details of our inclusion criteria were as follows: (1) evaluation of the polymorphism of *PSAP* rs4747203, rs885828, rs749823 and rs142614739 on PD risk; (2) well-designed case–control studies using similar research methods (with strict case selection, international diagnosis standard of PD, and appropriate statistical methods); (3) with sufficient published data to estimate an OR with 95% CI; (4) written in English and (5) containing detailed and useful genotype frequencies or alleles frequencies. Studies without clear and full information of samples were excluded. Two reviewers extracted information from all eligible publications independently, according to the above inclusion criteria. The sample size of each included sizes are listed in the Supplementary Table S3.

The risk of PD associated with *PSAP* rs142614739, rs4747203, and rs885828 was estimated for each study by ORs with 95% CI. For all studies, we estimated the association under the allele contrast model due to the availability of the published data. The Q-statistic was used to investigate the degree of heterogeneity between the trials, and a *p* value of more than 0.10 for the Q-test indicated a lack of heterogeneity among studies. We used the mixed-effect model, combining common effects and random effects from each of the studies based on the DerSimonian and Laird (DL) method. All of the meta-analyses were performed with R “meta” package^29^.

### Supplementary Information

Supplementary Information 1.

## Data Availability

The genotype data generated in NTUH during the current study are available from the corresponding author upon reasonable request. The summary statistics in other studies included in the meta-analysis are available from the published article.

## Abbreviations

AD: Autosomal dominant
AR: Autosomal recessive
DL: DerSimonian and Laird
EOPD: Early-onset Parkinson’s disease
FPD: Familial Parkinson’s disease
H&Y: Hoehn and Yahr
OR: Odds ratio
PD: Parkinson’s disease
PSAP: Prosaposin
95% CI: 95% Confidence interval

## References

[1] Bloem BR, Okun MS, Klein C (2021). Parkinson's disease. Lancet.

[2] Chu YT, Tai CH, Lin CH, Wu RM (2021). Updates on the genetics of Parkinson's disease: Clinical implications and future treatment. Acta Neurol. Taiwan.

[3] Klein AD, Mazzulli JR (2018). Is Parkinson's disease a lysosomal disorder?. Brain.

[4] Sardi SP (2017). Glucosylceramide synthase inhibition alleviates aberrations in synucleinopathy models. Proc. Natl. Acad. Sci. USA.

[5] Lee JS (2019). Arylsulfatase A, a genetic modifier of Parkinson's disease, is an alpha-synuclein chaperone. Brain.

[6] Oji Y (2020). Variants in saposin D domain of prosaposin gene linked to Parkinson's disease. Brain.

[7] Kishimoto Y, Hiraiwa M, O'Brien JS (1992). Saposins: Structure, function, distribution, and molecular genetics. J. Lipid Res..

[8] Tamargo RJ, Velayati A, Goldin E, Sidransky E (2012). The role of saposin C in gaucher disease. Mol. Genet. Metab..

[9] Cesani M (2016). Mutation update of ARSA and PSAP genes causing metachromatic leukodystrophy. Hum. Mutat..

[10] Chao YX (2021). Association analysis of PSAP variants in Parkinson's disease patients. Brain.

[11] Facchi D (2020). Saposin D variants are not a common cause of familial Parkinson's disease among Italians. Brain.

[12] Lin ZH (2021). PSAP intronic variants around saposin D domain and Parkinson's disease. Brain.

[13] Chen YP (2021). Genetic analysis of prosaposin, the lysosomal storage disorder gene in Parkinson's disease. Mol. Neurobiol..

[14] Richards S (2015). Standards and guidelines for the interpretation of sequence variants: A joint consensus recommendation of the American College of Medical Genetics and genomics and the association for molecular pathology. Genet. Med..

[15] Quan W (2021). Identification of potential core genes in Parkinson's disease using bioinformatics analysis. Parkinsons Dis..

[16] Sun Y (2013). Tissue-specific effects of saposin A and saposin B on glycosphingolipid degradation in mutant mice. Hum. Mol. Genet..

[17] Sosero YL (2020). Lack of evidence for genetic association of saposins A, B, C and D with Parkinson's disease. Brain.

[18] Nalls MA (2019). Identification of novel risk loci, causal insights, and heritable risk for Parkinson's disease: A meta-analysis of genome-wide association studies. Lancet Neurol..

[19] Singh RK, Cooper TA (2012). Pre-mRNA splicing in disease and therapeutics. Trends Mol. Med..

[20] Song J (2012). PROSPER: An integrated feature-based tool for predicting protease substrate cleavage sites. PLoS One.

[21] Avenali M, Blandini F, Cerri S (2020). Glucocerebrosidase defects as a major risk factor for Parkinson's disease. Front. Aging Neurosci..

[22] Smigielski EM, Sirotkin K, Ward M, Sherry ST (2000). dbSNP: A database of single nucleotide polymorphisms. Nucleic Acids Res..

[23] Henrie A (2018). ClinVar miner: Demonstrating utility of a Web-based tool for viewing and filtering ClinVar data. Hum. Mutat..

[24] Thompson AE (2016). The influence of gender and other patient characteristics on health care-seeking behaviour: A QUALICOPC study. BMC Fam. Pract..

[25] Hughes AJ, Daniel SE, Kilford L, Lees AJ (1992). Accuracy of clinical diagnosis of idiopathic Parkinson's disease: A clinico-pathological study of 100 cases. J. Neurol. Neurosurg. Psychiatry.

[26] Lin CH (2019). A clinical and genetic study of early-onset and familial parkinsonism in Taiwan: An integrated approach combining gene dosage analysis and next-generation sequencing. Mov. Disord..

[27] Wei CY (2021). Genetic profiles of 103,106 individuals in the Taiwan Biobank provide insights into the health and history of Han Chinese. NPJ Genom. Med..

[28] Papadopoulos JS, Agarwala R (2007). COBALT: Constraint-based alignment tool for multiple protein sequences. Bioinformatics.

[29] Balduzzi S, Rucker G, Schwarzer G (2019). How to perform a meta-analysis with R: A practical tutorial. Evid Based Ment. Health.

