# Supplementary material for: Prosaposin variants in sporadic, familial, and early-onset Parkinson's disease: a Taiwanese case–control study and meta-analysis
Source: Sci Rep. 2024 Jan 26;14:2225. doi: 10.1038/s41598-024-51646-y (PMC10817969; doi:10.1038/s41598-024-51646-y)

**Supplementary Tables and Figures**

**Table S1. Identified variants in patients with Parkinson’s disease in this study**

| **No.** | **Chromosome position*** | **Exon/Intron** | **cDNA alteration** | **Amino acid change** | **Variant Type** | **rs number** | **TWB frequency** | **GnomAD EAS** | **SIFT** | **Polyphen** | **ACMG pathogenicity** |
| --- | --- | --- | --- | --- | --- | --- | --- | --- | --- | --- | --- |
| 1 | chr10:71829016 | exon 5 | c.437C>A | p.A146E | Missense | NA | 0 | 0 | 0.02 | 1 | variant of uncertain significance |
| 2 | chr10:71825871 | exon 7 | c.743A>G | p.Y248C | Missense | rs2133040490 | 0 | 0 | 0.02 | 1 | likely pathogenic |
| 3 | chr10:71821915 | exon 8 | c.870G>T | p.K290N | Missense | NA | 0 | 0 | 0.34 | 0.295 | likely benign |
| 4 | chr10:71819853 | exon 10 | c.1053G>T | p.K351N | Missense | NA | 0 | 0 | 0.34 | 0.08 | likely benign |
| 5 | chr10:71819125 | intron 11 | c.1351-14A>G | NA | Intronic | rs4747203 | 0.622 | 0.345 | NA | NA | NA |
| 6 | chr10:71818746 | intron 12 | c.1432-22C>T | NA | Intronic | rs885828 | 0.3784 | NA | NA | NA | NA |
| 7 | chr10:71818948- 71818955 | intron 12 | c.1431+78_1431+83del | NA | Intronic | rs142614739/  rs74733861 | NA | 0.387 | NA | NA | NA |
| 8 | chr10:71818915 | intron 12 | c.1431+116C>T | NA | Intronic | rs749823 | 0.2346 | 0.026 | NA | NA | NA |

* Position on Genome Reference Consortium human genome build 38 (GRCh38).

*ACMG*, American College of Medical Genetics; *GnomAD EAS*, Genome Aggregation Database East Asia; *TWB*, Taiwan Biobank; *NA*, Not applicable.

**Table S2. Primer sequence.**

| Exon | Forward (5’-3’) | Reverse (3’-5’) |
| --- | --- | --- |
| exon 1 | CTCTGGACGGCTTTGGG | CTTGCCAACTAGGGCAGG |
| exon 2 | CTCAAGGACAGTCGCCCTC | AAAAGTGCTCTGTCAATATGGC |
| exon 3 | AAAGGCCATGGGGAGTTC | TCAGGCCTACACCATTCCTC |
| exon 4 | AGCTATTGAGGGCCTCTTGC | TGTAGCAAAATGCTGTTGAGG |
| exon 5 | AGAGGGACTAATTCAGAGGCAC | AGTCACGCACAGGGATAAGC |
| exon6 | CTTAGGAACTGGTCAGCAAGTG | GTCTGAACGCCCTACTCCAG |
| exon 7 | GACACTGTTGGAAATGCTGG | TTTAGCCCAATTCAGCACTC |
| exon 8 | GAGGTAGCCTTGACCTGGG | GGAACCGAAAGAAACAAGTGAC |
| exon 9 | TGGTCTCTGTGTCCCCTTTC | TCTCTCCTGTGGGACTTTCC |
| exon 10-11 | CCCACCATTGACTCATTTCC | CCCACCATTGACTCATTTCC |
| intron 11-12 | AGGGAACAGTGTATCCAGGGCCTG | AAAAGCAGGGTGGAGAGTTGATCAC |
| exon 12-13 | AGGGAACAGTGTATCCAGGG | AAAAGCAGGGTGGAGAGTTG |
| exon 14 | CCTGTTTTGGGGTGATATCTG | AGATCTGTGCGTTCATTCCC |

**Table S3. Sample size of included studies in the meta-analysis**

| Author | Year | PD case | Controls |
| --- | --- | --- | --- |
| Oji et al.^6^ | 2020 | 440 | 9,949 (gnomAD) |
| Chen et al.^13^ | 2020 | 700 | 9,976 (gnomAD) |
| Facchi et al.^11^ | 2020 | 366 | 3,538 |
| Sosero et al.^17^ | 2020 | 4,132 | 4,470 |
| Lin et al.^12^ | 2021 | 487 | 482 |
| Chao et al.^10^ | 2021 | 1,714 | 2,048 |
| Nalls et al.^18^ | 2019 | 37,688 | 1,400,000 |
| Kuo et al. | 2023 | 887 | 711 |

**Figure S1. Multiple sequence alignment of two exonic variants across varied species.** Multiple species alignment of the *PSAP* gene indicated that the mutations at p.A146E (A) and p.Y248C (B) are both located in evolutionarily conserved regions.


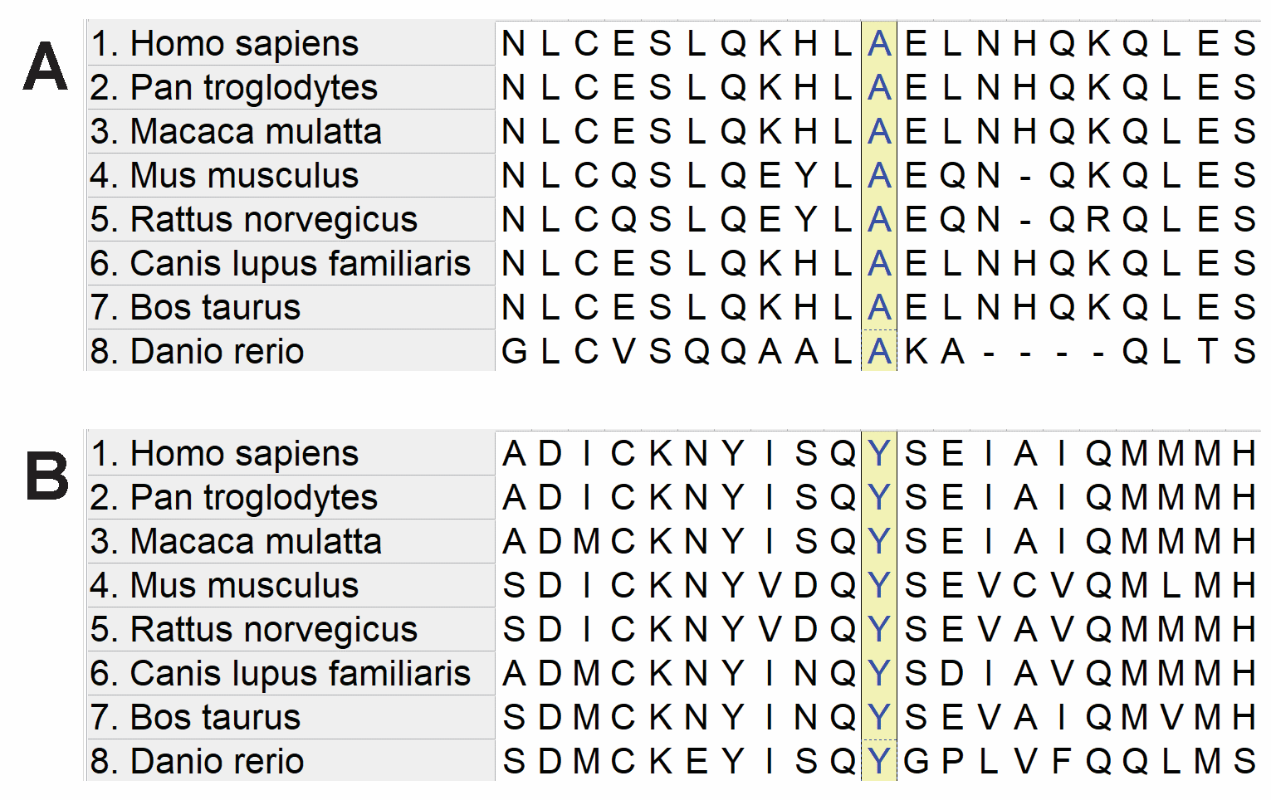

Supplement: Supplementary file 1 — Supplementary Information 1. [file 41598_2024_51646_MOESM1_ESM.docx]
